# Editorial: Stevens Johnson syndrome: past, present, and future directions

**DOI:** 10.3389/fmed.2024.1383891

**Published:** 2024-03-07

**Authors:** Hajirah N. Saeed, Robert Micheletti, Elizabeth J. Phillips

**Affiliations:** ^1^Department of Ophthalmology, Illinois Eye and Ear Infirmary, University of Illinois Chicago, Chicago, IL, United States; ^2^Department of Ophthalmology, Loyola University Medical Center, Maywood, IL, United States; ^3^Department of Dermatology, Perelman School of Medicine, University of Pennsylvania, Philadelphia, PA, United States; ^4^Center for Drug Safety and Immunology, Vanderbilt University Medical Center, Nashville, TN, United States; ^5^Institute for Immunology and Infectious Diseases, Murdoch University, Murdoch, WA, Australia

**Keywords:** SJS, TEN, HLA, complications, community, testing, diagnosis

Stevens Johnson syndrome/toxic epidermal necrolysis (SJS/TEN) is a rare immune-mediated mucocutaneous disease with a global incidence of up to 12 cases per million population annually (1). SJS/TEN cumulative hospitalization cost is ~$128 million per year, and mortality rates can exceed 50% in the immunocompromised and elderly (2). There are still many gaps in knowledge about the pathogenesis of SJS/TEN and, hence, in the ways to optimize prevention, earlier diagnosis, targeted acute treatment and long-term management. (3).

In this Research Topic, a wide breadth of novel data and new insights is presented by researchers globally, which reflects the current collaborative work that is ongoing to eliminate the morbidity and mortality of this devastating and life-threatening disease. As a direct result of this collaboration, the SJS/TEN biennial conference was established in 2017 that promoted patient and community involvement (4). The 3rd biennial conference, SJS/TEN 2021 (Marks et al.), brought together 428 international scientists and 140 survivors and family members. The goal of the meeting was to brainstorm strategies to support the continued growth of an international SJS/TEN research network, bridging science and the community. The community workshop section of the meeting focused on eight primary themes: mental health, eye care, SJS/TEN in children, non-drug induced disease, long-term health complications, new advances in mechanisms and basic science, managing long-term scarring, considerations for skin of color, and risks of COVID-19 vaccines. This meeting has since been followed by SJS/TEN 2023 “Bringing Science to All” in August 2023 that tackled an overarching theme of overcoming geographic, social, and economic barriers and disparities, aiming to be inclusive of all populations. Many of these same contributors from the 2023 meeting who presented novel data are represented in this Research Topic.

Over the last decade, there have been considerable insights on immunotherapy and immune-checkpoint-inhibitor-therapy-induced SJS/TEN as well as novel methods to improve the diagnosis, management, and mortality risk stratification of SJS/TEN, and organ-specific pathology.

Chen et al. and Kurian et al. highlight SJS/TEN occurring in association with novel agents: the epidermal growth factor receptor tyrosine kinase inhibitor toripalimab and the α-specific PI3K inhibitor alpelisib. Kuo et al. (5) highlight the addition of severe cutaneous immune related adverse events associated with immune checkpoint inhibitors and highlight knowledge and evidence gaps related to diagnosis and treatment. The presentation can vary from SJS mimickers such as lichenoid and autoimmune bullous disorders to presentations more in keeping with traditional SJS/TEN. In their comprehensive 40 year literature review, Wang et al. identify the demographics, clinical course, and mortality risk of 379 drug culprits across four common classes associated with SJS/TEN.

While notable recent advances in SJS/TEN have made risk prediction and prevention possible for some causative factors, many challenges still exist in the scoring and documentation of the severity of SJS/TEN, which has not been standardized to be reproducible across individual cases and treatment centers. This phenotyping is a pre-requisite for engaging in further studies, particularly clinical trials, to assess the efficacy of therapeutics and other interventions. Shareef et al. examine the predictive value of a random forests classifier for mortality compared with SCORTEN, the most commonly used mortality risk prediction tool. SCORTEN requires calculation of total body surface area detached, which is subject to considerable observer error and variability. In their model, which used only routine laboratory information, the top five predictors of mortality were RBC count, total bilirubin, prothrombin time, WBC count and RBC count.

Beyond mortality prediction, however, other novel diagnostic and staging systems include medical photography to monitor progression and treatment response. Dobry et al. summarize the state of current clinical assessment and scoring tools and highlight the need for standardized approaches to measure cutaneous involvement. Lehloenya highlights the need for reproducible endpoints in clinical studies and consideration for innovative approaches such as use of biological markers, artificial intelligence, and imaging approaches (e.g., PET/CT scan) to monitor progression and therapeutic response.

In adults, >80% of SJS/TEN is caused by a small molecule drug. SJS/TEN is also marked by tissue specificity. Hence, “blood tests” and *ex vivo*/*in vitro* tests to define drug causality have been challenging. Copaescu et al. comprehensively review the current state of *in vivo* and *ex vivo*/*in vitro* diagnostic tools of potential use in severe cutaneous adverse drug reactions, including SJS/TEN, to aid diagnosis and drug causality. The sensitivity of both patch testing and *in vitro*/*ex vivo* testing in SJS/TEN was dependent on the culprit medication and was lower (< 50%) for SJS/TEN than for drug reaction with eosinophilia and systemic (DRESS). Genetic testing has been posited in the past as a screening tool for the prevention of SJS/TEN or DRESS where this is a well-established association. When an HLA allele is distinct for a specific medication, genetic testing, along with patch testing and *in vitro*/*ex vivo* testing, may also aid in diagnosis.

Although there is agreement on harmonized supportive care and comprehensive ophthalmological, cutaneous, and urogynecological management in an acute critical care setting, the long-term effects of acute SJS/TEN and its management on long-term complications are largely unknown. DenAdel et al. present a retrospective chart review of 77 biopsy-supported female patients with SJS/TEN treated at a single center. They were able to measure a positive impact by protocolizing acute management that guided gynecological consultation and appropriate treatment of vulvovaginal disease.

Although there has been much progress in SJS/TEN research, many gaps exist. In addition to genetic factors, more study is needed on the social determinants of health that can drive medication utilization and impact risk in disadvantaged populations (6, 7). Further study of tissue specific responses will help define markers for earlier diagnosis and targeted treatment. A system that ensures that care does not end at hospital discharge is also crucial to providing necessary medical and psychological support and to prevent and support long-term complications such as visual impairment and blindness (8). Particularly important is documentation and a health “passport” to avoid exposure to culprit medication(s) and ensure drug safety for the future.

## Author contributions

HS: Writing – review & editing, Writing – original draft, Conceptualization. RM: Writing – review & editing, Conceptualization. EP: Writing – review & editing, Writing – original draft, Conceptualization.
